# Service-Learning with College Students toward Health-Care of Older Adults: A Systematic Review

**DOI:** 10.3390/ijerph16224497

**Published:** 2019-11-14

**Authors:** Pedro-Jesús Ruiz-Montero, Oscar Chiva-Bartoll, Celina Salvador-García, Ricardo Martín-Moya

**Affiliations:** 1 Department of Physical Education and Sport, Faculty of Education and Social Sciences, Campus of Melilla, University of Granada, 52071 Melilla, Spain or; 2 Faculty of Education, University of Málaga, 29071 Málaga, Spain; 3 Department of Education and Specific Didactics, Faculty of Humanities and Social Sciences, Universitat Jaume I, 12005 Castellón, Spain; 4 Body Expression Area, Education School, University of Granada, 18071 Granada, Spain

**Keywords:** aging, education method, higher education, healthy lifestyle, community learning

## Abstract

Service-Learning (SL) has become a teaching methodology that promotes social and personal skills while helping groups in need and at risk of social exclusion. This paper is a systematic review of the literature on SL experiences and research on college subjects in the area of health-care promotion in settings for older adults. After an exhaustive search, 43 peer-reviewed publications were classified according to frequency and geographical distribution, sample and duration of the programs, research methodologies, data collection instruments used, and main outcomes investigated. The results indicate that the research methodologies used tended to be qualitative and mixed, while the variety of samples and duration of interventions was very broad. The instruments used were mainly interviews and questionnaires, and the programs were developed specially in the United States of America. The groups receiving most SL were healthy older adults and older adult populations with aging disabilities and illnesses. The articles in the present review highlight that SL can have a positive effect on older adults’ health promotion and can enhance their community participation.

## 1. Introduction

The World Population Ageing report [1] concluded that, by 2050, the global population aged 60 years or over will reach nearly two billion. Moreover, the number of people older than 80 is growing faster than the number of people in their 60s. The population of older adults experiences an objective decrease in physical abilities that may be accompanied by psychological disorders and a loss of social and affective relationships [2]. That is, in general, older adults’ health level and quality of life are impaired.

This might be alleviated with the increase of general knowledge related to gerontology, as well as supporting issues related with well-being, active aging, and physical and mental health in aged people [3]. Intergenerational experiences are often used in gerontological education because they are believed to improve older adults’ quality of life together with students’ understanding of aging [4]. At this point, as a note of caution, it is important to warn that colleges should increasingly reflect the needs, concerns, and intentions of society. Current college education must strive to create the optimum conditions in which young people acquire the appropriate skills to support a sustainable society. This scenario is an optimal field for students to acquire varied resources to create a fairer and more egalitarian society with and for those who need it most [5]. This fact justifies and requires the promotion of service-learning (SL) in different settings, such as among older adults or other groups in need.

SL is a pedagogical methodology that establishes a genuinely operational relationship between theory and practice and provides students with the opportunity to learn while contributing to the community. SL combines learning and community service, based on the application of skills and reflective understanding of curriculum content in real contexts, experientially, and with the aim of improving students’ critical skills while offering a social benefit [6].

The increase in SL has roused great interest in educators at all levels and academic disciplines. Consequently, a multitude of studies examining its impact have been implemented, particularly in higher education [7]. Due to this proliferation, the need to better understand SL at the college level is urged by all agents committed to its implementation. Previous studies, such as the meta-analyses of Conway et al. [8] and Warren [9], have offered a panoramic overview of SL in terms of research and implementation. It remains necessary, however, to provide specific analyses from each discipline and educational level [7].

SL requires great deal of effort and attention in the specific field of health-care promotion and community participation. It is important to note that the American College of Sports Medicine highlights the need to have some basic knowledge in working with different collectives while focusing on health promotion and community participation [10]. For this reason, it is necessary to value and be aware of the possibilities and insights of SL implemented among older adults, a population group that requires special attention, through the analysis of previous applications.

This study presents a systematic review of the literature focused on higher education SL aimed at achieving benefits associated with health promotion and community participation among older adults. There is substantial literature on the beneficial impact of volunteering on older adults, much of which helps older adults [4]. However, volunteering and SL are not the same thing. Consequently, studies on volunteering are not completely valid to describe in depth the experiences of SL participants, as well as other features of this pedagogical methodology.

Concerning the conjunction of work among older adults and SL, Roodin et al. [11] have presented a review of the general literature focused on intergenerational SL. However, their approach is very wide and does not focus specifically on the field of health-care promotion. Indeed, the authors suggest the need for future specific studies. Their study also highlights the gap in measuring SL effects, the paucity of appropriate instruments to assess its effects, and the differing operational definitions of SL.

The present review is therefore structured around the following descriptive categories: Frequency with which SL articles on older adult populations are published, geographical distribution of papers, sample and duration of the programs, research methodologies employed, data collection instruments used, and the main outcomes investigated. Knowing the main advantages and disadvantages involved in these dimensions before starting a program can be critical for SL success.

## 2. Methods

Because there have been some critiques of previous reviews that have not adopted a transparent and reproducible search protocol [12], we were committed to following a systematic and explicit method [13]. The present literature review used the integrative review method, including the following steps: Using the process of problem identification, articulating search terms, formulating inclusion criteria, developing the literature search, screening the articles based on the inclusion criteria, analyzing the data, and finally synthesizing and reporting the findings. In addition, this systematic review was performed according to the Preferred Reporting Items for Systematic Reviews and Meta-Analyses (PRISMA) guidelines and its checklist was used to apprise the quality of the study.

### 2.1. Search Strategies

After identifying the problem, two main search strategies were used. The first step consisted of a search in two of the most respected databases in the field of social sciences and education: (ISI)-Thomson Reuters and Scopus-Elsevier. From these, we considered all peer-reviewed publications from the 21st century, that is, between 2001 and July 2019. Due to the variety of the terminology used in the literature, we sought to identify multiple terms to capture the intersection of ideas for our review. All of the publications therefore had to contain at least one term related to the following concepts: ‘Service-learning’ (service-learning, service learning, community learning, experiential education, experiential learning); ‘health’ (health, healthy habits, healthy lifestyle, wellness, quality of life); ‘higher education’ (university, college, higher education); and ‘older adults’ (older adults, elderly, elders, senior adults, senior). The search strings consisted of a combination of the terms for each concept, which were scanned for in titles, keywords, and abstracts.

Finally, as a second step, the reference lists of all retrieved articles were reviewed to identify the possible existence of other interesting publications on the subject of SL in the field of health and higher education with older adults. The reason for choosing articles from the last 19 years was to highlight selected existing studies and trends from the current century.

### 2.2. Inclusion Criteria

The selection of the literature to be reviewed was based on four general criteria: Quality, relevance, educational level, and topicality. Papers that did not meet these criteria were excluded from further consideration. Regarding quality and relevance, the first filter was the academic nature of the database where the articles were found and the accomplishment of the PRISMA literature search procedure. The format of each item also had to meet the standardized scientific criteria by being empirical, narrative, or reasoned, with well-defined objectives, methodologies, and designs containing valid and recognized studies. Regarding educational level and topicality, the search was directed solely at SL programs in the field of health care that had college students as the learners and older adults as service receivers.

The results of the search for the selected articles should also respond to the type of research and/or application carried out through the SL experience in a field related to the subjects of health with older adults, excluding internships, scholarships, or volunteering. A team of four researchers applied the inclusion criteria. To ensure reliability in the application of the criteria, this research occurred after a one-day moderation. For the most part, the decision to include articles was relatively straightforward, because there was clear evidence of whether the papers were aligned (or not) with the review’s purpose.

## 3. Results

**Figure 1:**
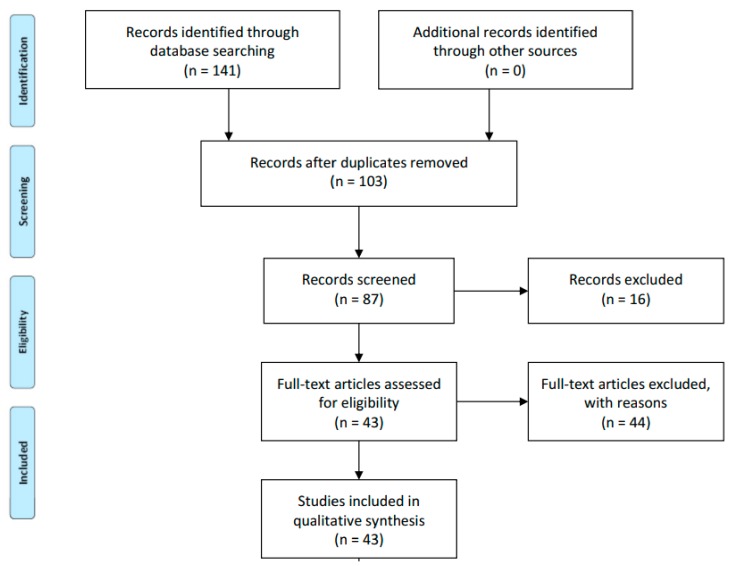
Literature search methodology using the Preferred Reporting Items for Systematic Reviews and Meta-Analyses (PRISMA) framework.

The initial search within the two databases yielded 753 potentially relevant articles. After reviewing title, keyword, and abstract information, the sample of potential articles was reduced to 141. No other interesting publications were found among their reference lists. There were 38 repeated publications, 16 records were excluded because they were abstracts that did not provide enough data, and 44 articles did not meet the inclusion criteria, leaving 43 articles for review. Each of the papers in the final sample was classified according to the following descriptive categories: Frequency and geographical distribution, sample and duration of the program, research methodology, data collection instruments used, and main outcomes investigated. The PRISMA literature search methodology is graphically represented in Figure 1.

### 3.1. Frequency of Impact Publications and Geographical Distribution

**Figure 2:**
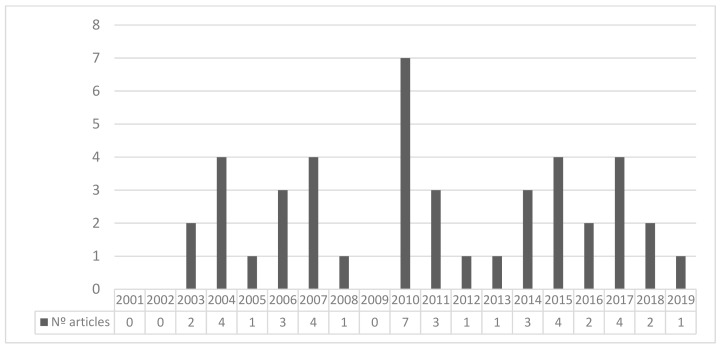
Number of articles by year of publication. 2019 review includes the only paper published until July.

According to the publication dates, the results do not show clear patterns in terms of frequency (Figure 2). The most prolific year was 2010 (19.44% of the sample) followed by 2004, 2007, 2015, and 2017 with four publications each, as may be observed in Figure 2. More than half (52.78%) of the whole sample was published in these four years. The years 2001, 2002, and 2009 were not represented in the present review. The remaining years fluctuated between one and three articles that met the inclusion criteria. Despite the variation in the number of articles per year, their publication appears to remain noticeably constant after 2003, a year that seems to be pivotal for research on the subject.

Another aspect that should be noted is the geographical distribution of the programs, because this will reveal the world’s leading research centers for college SL involving health promotion among aged people. The majority of articles (83.7%) were developed in the United States. Apart from this, the rest of the sample does not show more relevant patterns.

### 3.2. Sample and Duration of the SL Programs

SL programs may vary widely depending on features such as aim, service learners, scope of action, or service receivers. We may note a disparity in both the number of students giving service and the number of elders receiving it. In terms of the former, this number of individuals ranges from 16 in the works of Fruhauf et al. [14] and Singleton [15] to 509 in Hegeman et al. [16]. These numbers include students from a range of degree programs, such as nursing education, psychology, physical therapy, or pharmacology. SL can be developed embedded within a university subject or program, but it can also be proposed as an extracurricular activity. When it is part of a university curricular program, it can be implemented through projects, academic courses, workshops, or single lessons, and 23.3% of the sample (10 articles) refers to this [3,14,17,18,19,20,21,22,23,24], whereas 33 articles consider SL as extra academic training in the university (76.7%). Looking at the samples of older adults, the number of people receiving service is quite variable, as are the group profiles, which can consist of healthy older adults or people suffering different illnesses or any type of social disadvantage.

In terms of the length of the programs and sessions involved in the investigations, it is worth noting the complexity of classification and analysis due to the varying information provided in each text. The majority of papers present studies or experiences that are part of larger programs, as in the cases of Timmermans et al. [24] or Lynch et al. [25]. Among those articles that detail the time of service given, the most repeated was one semester [14,15,17,18,22,25,26,27,28,29,30,31] or 20 hours [32,33]. The shortest SL interventions or experiences were three hours [34], one day [35], or two clinical days [36], whereas the longest were three-year projects [3,24,37,38].

### 3.3. Research Methodology

Several differences appeared among the research methodologies. In this sense, Creswell [39] differences three major approaches to research: (1) Quantitative approach in which the researcher primarily uses positivist claims for developing knowledge and yields statistical data, (2) qualitative approach in which the investigation is based primarily on constructivist or advocacy perspectives and attempts to develop themes from the data, and (3) mixed methods approach, which is based on pragmatic grounds and involves gathering both quantitative and qualitative information.

Regarding our sample, 34.8% of the publications employed a qualitative method, whereas 13.9% used a quantitative approach. In addition, nearly half of the sample (46.5%) used a mixed approach. The remaining two papers (4.8%) do not evince any type of methodology because they are devoted to a description of experiences [40] and a pilot study [41].

Moreover, in this field it is important to analyze whether the effects of SL remain or disappear over time for both populations, service providers and service receivers. In this sense, only four out of 43 articles (9,3%) used long-term designs.

### 3.4. Data Collection Instruments

The instruments used to gather the data are another relevant aspect. A total of 30 articles employed surveys and/or questionnaires. Specifically, there were four articles that only used standardized questionnaires [14,29,42,43], and only two that applied questionnaires to a control group [17,23], while Sookhai et al.’s study [44], on the other hand, used a non-standardized questionnaire. A standardized survey is used by Faria et al. [45], whereas ten articles used non-standardized surveys. Moreover, 20 articles combined questionnaires and surveys. Only one study, that by Beling [17], used a standardized and non-standardized data collection instrument and it there was also a systematic review [28]. Reflective logs are used as evaluation and feedback by students in five articles [27,34,35,40,46]. Finally, two articles analyze the SL program and provide some useful recommendations for future implementations based on participatory action research or similar approaches [46,47].

### 3.5. Main Outcomes Investigated

The main objectives addressed by researchers can be principally divided into two areas of interest: The effects of SL on the students who provide the service and the impact on the older adults who receive it. The majority of the articles (65.1%) generated results related to the college students. A wide range of aspects was considered, for example, Andreoletti and Howard [46], Augustin and Freshman [33], and Lokon et al. [29] concentrated on the increase of students’ positive perceptions; Kohlbry and Daugherty [35] analyzed the development of their cultural awareness, knowledge, and skills; Chen [46], D’Abundo et al. [27], and Giné-Garriga et al. [48] observed students’ practical experience; Neill et al. [43] and Neal et al. [49] examined the improvement of students’ perception of professional competence; and, finally, Gazsi and Oriel [34] investigated the integration of course content through applied SL. For the rest of the sample, only four papers (9.3%) focused on aspects such as older adults’ perceptions or changes, experiences, learning, thoughts, feelings, or relationships [4,14,36,44].

In addition, 25.6% of the sample examines both older adults and college students [15,16,19,22,25,26,30,31,38,41]. On the one hand, focusing on college students these articles address variables such as the conception of aging, practical learning about their professional training, enthusiasm to serve older adults, and knowledge of public health essential services. On the other hand, the main variables analyzed in older adults refer to the prevention and management of falls, learning to apply some basic health treatments, and computer and recycling related learning.

At this point, it is important to note that only four studies used long-term designs (4.6%), two analyzed community service involvement and positive attitudes on students [32,33], and two referred to variables such as learning to prevent falls [50], and social engagement and positive thoughts in older adults [25]. All of them reported positive results.

It is noteworthy that one of the articles examines the particular effects of SL on the program as a pedagogical process [37], and two articles [46,48] analyze the programs and provide recommendations for both, the implementation and the investigation of SL. For its implementation they refer to aspects such as involvement and cooperation with a suitable community organization, setting clear service purposes, a detailed curriculum design that is prepared in advance and aligns fully with the service activities planned, service skill training for students, immediate support and discussion during the service process, and space and time for subsequent reflection and comments. To improve research, Chen’s study [46] recommends the inclusion of a control group in order to provide a more valid insight into the impact of the SL investigations. In this regard, the present review only found two articles (2.3%) that used control group in their designs [17,23].

Finally, seven articles (16.3%) refer to the general satisfaction with the SL program, four of them (9.3%) indicate that college students were satisfied, whereas three (7%) refer to the older adults’ general satisfaction. The synthesis of all of papers that met the inclusion criteria for this review is presented in Table 1.

## 4. Discussion

According to the data obtained in this systematic review, higher education SL has been used on a number of occasions for health-care promotion among older adults and to encourage community participation. The experiences that constitute the sample enabled both college students and older adults to overcome stereotypes and biases to gain multigenerational perspectives, which triggered an improvement of mutual understanding and cohesion while favoring older adults’ wellness. However, there are many alternatives through which this area of study might be improved by future researchers and to provide better SL programs addressing issues related with the health of aged people.

The analysis of the literature shows that the frequency with which SL articles on older adult populations have been published does not follow a gradual development, although it is a recurring topic since the beginning of the 21st century. The programs described may vary widely depending on features such as study methodology, duration and intensity of the SL program, location, service learners or service receivers, and scope of action. There is great diversity in terms of program length, but the most repeated ones were programs lasting one semester and providing 20 hours of service, which is a result consistent with the reviews made by Chiva-Bartoll et al. [56] or Copaci and Rusu [57]. This might reinforce future volunteering because Antonio et al.’s study [58] associates the high attendance of college students in SL programs with future repetitions as volunteers.

Regarding the type of research methodology employed in settings that opt for SL as a way to promote health care among older adults, there is a clear predisposition towards qualitative or mixed approaches. This result is in line previous literature on SL [59,60] and responds to the need for collecting relevant data from the participants to control for confounders [61]. Related to this aspect, another result of this review is the predominant use of surveys or questionnaires, also in agreement with the review carried out by Eidson et al. [61], who found that authors tended to develop their own instruments for their SL investigations.

In the pedagogical context it is important to know the lasting effects of the SL programs implemented. This systematic review found four long-term studies published, two referring to attitudes of college students [32,33], one referring to social engagement and positive thoughts in older adults [25], and one referring to the improvement in the prevention of falls in older adults [50]. In this vein, this research highlights the need to take into account more longitudinal studies, with the aim of deepening not only in the analysis of perceptions and attitudes of both, students and older adults, but also in permanent changes and behavior.

The main issues investigated in the papers analyzed in this systematic review may be classified into two categories: Those relating to students and those relating to older adults. Some studies reported positive results in learning outcomes related to the personal perceptions of students, as well as the acquisition of professional skills. The benefits to college students are positive and well documented [11]: The knowledge of the consequences of aging among older adults [62], specific geriatric issues [14], or the perceptions of professional cooperation [43] increase in college students. The outcomes for older adults are mainly focused on their perceptions and general satisfaction [16,25,31,38], although some articles also refer to the prevention and management of falls [50], impact of activity trackers [43], and computer [41] and recycling [27] related learning.

In general, the majority of published results report better social stimulation and understanding of young people after a SL intervention and the general satisfaction of older adults. In addition, a program managed by Zucchero [4] with a pairing of independent older adults and volunteer students showed an improvement in mutual relationship formation and positive experiences in the aged. The results clearly show that SL may be an optimal way to promote intergenerational social cohesion and promote older adults’ health care [63,64].

The literature review shows that the United States is a country with a great tradition and proven experience in SL. Indeed, the term SL was first used in the United States by Ramsay et al. in 1967 [65], and the United States has formally incorporated SL into its educational systems. It is important to highlight that there is a worrisome relationship between health and socioeconomic, demographic, or cultural factors in the United States [66]. Health problems, physical limitations, and chronic diseases caused by sedentary lifestyles can be lessened and reduced in developed countries thanks to physical exercise [67] through SL interventions [3]. For instance, the health system in Australia, the United Kingdom, the United States [68], or Spain [69] are also costly due to indirect effects such as primary care setting, medication, treatments, or surgeries. Most of them are related to the aging process [68,69,70]. Thus, health-care promotion in SL interventions could help to alleviate the monetary burden for the government and citizens.

According to the political dimension of civic activities, college students might become more quickly and profoundly aware of the benefits of voluntary service among older adults thanks to their own SL experience. The result may be a progressive increase of college students implementing SL with older adults and, consequently, developing social cohesion between these two groups of people. Another important aspect is the wide variety of differentiating aspects among SL programs and the scope of action in which are implemented [8,9], including the number of participants and the intensity and duration of the programs implemented. It can be concluded that, beyond the disciplinary scope and educational level, SL is a pedagogical method that can be adapted to give individual answers to the characteristics and needs of different groups of older adults, such as by focusing on health promotion, as well as different groups of college students, by focusing on their acquisition of a range of knowledge, skills, and abilities. This is fully in accordance with the review of Roodin et al. [11] concerning college students, intergenerational SL, and gerontology.

## 5. Conclusions

To conclude, consistent with Novak et al. [7] and Warren [9], the results of the current study suggest that SL has a positive effect on student learning outcomes, the gold standard when measuring pedagogical practices. However, we must not forget that this methodology provides a service that can be extremely useful for the receivers and, therefore, SL can have a positive effect on health promotion among older adults as well. This is an encouraging result for educators considering implementing an SL component in their courses or at their universities. SL programs focused on areas such as older adults’ health care (whether or not they include a research component) can be a particularly useful step in promoting positive attitudes toward working with older adults to promote social cohesion and improve community participation. However, further research is still needed to evaluate the outcomes of SL programs to facilitate efforts by faculty members to create optimal learning experiences and the finest service programs. According to this systematic review, many of the studies published in the field only analyze the perceptions and learnings of the college students during or just at the end of the SL program. In the future, it would be interesting to deepen on the effects on both, students and older adults. In addition, following the guidance of Bringle et al. [71], Billig and Waterman [72], and Waterman [73], it would be advisable to use long-term studies to clear doubts about the effects of SL in the long term. Finally, we should mention the limitations of the current study, because the generalizability of these results could be problematic due to the possibility that further unpublished research exists that could potentially affect the overview. We were also not able to gauge the quality of the SL experiences in each of the included studies.

## Figures and Tables

**Table 1:** Characteristics of the papers included in the review.

Source	Type of Study/Data Collection Instruments	Duration and/or Intensity	Number of Participant Students	Profile of Older Adults	Scope of Action	SL Program as Part of…	Geographical Distribution
Andreoletti and Howard (2018) [47]	Mixed: Questionnaires and surveys	3 semesters	50	Local assisted living community	Psychology	University program	Connecticut, USA
Arkin (2011) [32]	Qualitative: Non-standardized surveys	45 hours of SL, 8 semesters	111	Early-stage Alzheimer’s disease	Various university degrees	University program	Arizona, USA
Augustin and Freshman (2015) [33]	Qualitative: Non-standardized surveys	20 hours of SL, 10 week course	24	Healthy older adults	Nursing education	University program	California, USA
Beling (2003) [17]	Quantitative: Standardized and non-standardized questionnaires	1 semester	40	Healthy older adults	Physical therapy	University curricular program	California, USA
Bonner et al. (2007) [50]	Mixed: Questionnaires and surveys	3-month period	76	Healthy older adults	Nursing education	University program	Massachusetts, USA
Bullock (2017) [26]	Mixed: Questionnaires and surveys	1-semester project	19	Chronic illnesses and healthy older adults	Pharmacy	University program	Texas, USA
Butler and Baghi (2008) [42]	Quantitative: Standardized questionnaires	3-month period, 20 hours per week	28	Healthy older adults	Nursing, gerontology, and health science education.	University program	Fairfax, Virginia, USA
Chen (2018) [46]	Qualitative: Reflective log	6 weeks	31	Healthy older adults from rural community	Social gerontology	University program	Taiwan
D’Abundo et al. (2011) [27]	Qualitative: Reflective log	1-semester project	43	Healthy older adults	Nursing education	University program	North Carolina, USA
Dorfman et al. (2007) [18]	Mixed: Questionnaires and surveys	1-semester project	167	Healthy older adults	School of social work	University curricular program	Iowa, USA
Ellison and Radecke (2005) [28]	Qualitative: Systematic review	1-semester project	18	Advanced aging and terminal illnesses.	Various university degrees	University program	Pennsylvania, USA
Eskelinen et al. (2014) [37]	Qualitative: Non-standardized surveys	3-year project	60	Healthy older adults	Bachelor of social services	University program	Estonia, Finland
Faria et al. (2010) [45]	Qualitative: Standardized surveys	3-semester project	37	Healthy older adults	Bachelor of social work	University program	NY, USA
Fruhauf et al. (2004) [14]	Quantitative: Standardized questionnaires	1-semester project	16	Dementia	Aging or sociology of aging	University curricular program	Colorado, USA
Fuller et al. (2006) [40]	—: Reflective log	1 academic course	18	Older adults with disabilities and/or living alone	Nursing education	University program	South Carolina, Columbia, USA
Fusner and Staib (2004) [36]	Qualitative: Non-standardized surveys	2 clinical days of SL	40	Chronic illnesses or frail and healthy older adults	Nursing education	University program	USA
Gazsi and Oriel (2010) [34]	Qualitative: Reflective log	3 hours of SL	17	Frail and healthy older adults	Physical therapy	University program	USA
Giné-Garriga et al. (2019) [48]	Mixed: Questionnaires and surveys	8 workshops	26	Chronic illnesses or frail and healthy older adults	Physical therapy and sport sciences	University program	Spain and Scotland
Gustavson and Coppola (2006) [41]	—: Non-standardized surveys	14 weeks	23	Older adults with physical limitations	Various university degrees	University program	USA
Hegeman et al. (2010) [16]	Mixed: Questionnaires and surveys	4-semester project	509	Healthy older adults	Various university degrees	University program	Albany, NY, USA
Horowitz et al. (2010) [3]	Mixed: Questionnaires and surveys	3-year project	69	Healthy older adults	Bachelor of science in health sciences	University curricular program	NY, USA
Howell et al. (2017) [19]	Mixed: Questionnaires and surveys	80 hours per course	125	Older adults from underserved communities	Pharmacy	University curricular program	Texas, USA
Jorgenson et al. (2016) [51]	Mixed: Questionnaires and surveys	1-year program	-	Chronic illnesses with medical treatment	Pharmacy education	University program	Canada
Kalisch et al. (2013) [52]	Qualitative: Non-standardized surveys	12 hours of SL	102	Healthy older adults	Nursing education	University program	USA
Kim et al. (2003) [20]	Mixed: Questionnaires and surveys	12 hours of SL, 5-week project	49	Healthy older adults	Dietetics education	University curricular program	Ohio, USA
Kimzey et al. (2016) [53]	Mixed: Questionnaires and surveys	6-hour clinical day at each location and 1-hour post conference	94	Alzheimer’s disease	Baccalaureate education	University program	USA
Kohlbry and Daugherty (2015) [35]	Qualitative: Reflective log	1 day of SL	37	Homeless and people with chronic diseases	Nursing education	University program	México
Kolomer et al. (2010) [21]	Mixed: Questionnaires and surveys	2-semester project	83	Healthy older adults	Nursing and social work education	University curricular program	USA
Krout et al. (2010) [38]	Mixed: Questionnaires and surveys	3-year project	225	Stroke patients	Various university degrees	University program	USA
Lapp and Caldwell (2012) [22]	Mixed: Questionnaires and surveys	1-semester project	21	Healthy older adults	Nutrition and psychology education	University curricular program	NY, USA
Lokon et al. (2017) [29]	Quantitative: Questionnaires	1-semester project	156	Older adults with dementia	Gerontology, social work and education	University program	Florida, USA
Lynch et al. (2014) [25]	Qualitative: Non-standardized surveys	1 semester in 3 years of coursework	-	Healthy older adults	Various university degrees	University program	USA
Mathew et al. (2011) [23]	Mixed: Questionnaires and surveys	2-month course	60	Healthy older adults	Medical education	University curricular program	United Arab Emirates
Mobley-Smith et al. (2004) [54]	Mixed: Questionnaires and surveys	4-month project	64	Healthy older adults	Pharmacy education	University program	Illinois, USA
Neill et al. (2007) [43]	Quantitative: Standardized questionnaires	8-semester project	114	Older adults with medical treatment	Various university degrees	University program	Idaho, USA
Neal et al. (2017) [49]	Mixed: Questionnaires and surveys	Two weeks	149	Healthy older adults living in rural areas affected by natural disasters and civil war	Various university degrees	University program	Oregon, USA (students)Nicaragua (SL)
Oakes and Sheehan (2014) [55]	Mixed: Questionnaires and surveys	—	23	Healthy older adults	Various university degrees	University program	USA
Romack (2004) [30]	Qualitative: Non-standardized surveys	1 semester, 15–20 hours of SL	22	Frail older adults	Nursing education	University program	California, USA
Singleton (2007) [15]	Qualitative: Non-standardized surveys	30 hours of SL, 1 semester-project	16	Healthy older adults	Baccalaureate, social work education	University program	USA
Sookhai et al. (2015) [44]	Quantitative: Non-standardized questionnaires	7-week study	-	Healthy older adults	Computer Science and Information Systems education	University program	NY, USA
Timmermans et al. (2015) [24]	Mixed: Questionnaires and surveys	3-year project	387	Dependent older adults	Nursing education	University curricular program	France, England, Belgium, and the Netherlands
Underwood and Dorfman (2006) [31]	Qualitative: Non-standardized surveys	1-semester project	-	Healthy older adults	Nursing education	University program	USA
Zucchero (2010) [4]	Mixed: Questionnaires and surveys	1-semester project	-	Healthy older adults	Psychology education	University program	USA
